# Notes and Notices

**Published:** 1870-11

**Authors:** 


### NOTES AND NOTICES.

"CONTAGIOUS DISEASES ACTS," in reference to Women, from a Sanitary point of view. In four parts, by CHARLES BELL TAYLOR, M. D. F. R. C. S. E. London and Nottingham, England. This is a pamphlet circulated in the interests of humanity. All interested in the Social Evil will find its pages extremely suggestive.

LEE & SHEPPARD, of Boston, are among the most industrious and indefatigable publishers in the United States, and issue more new volumes every season than would have sufficed a continent a few years ago. Among the later books are, "Princes of Art—Painters, Sculptors and Engravers;" translated from the French by Mrs. S. D. URBINO; 337 pp., 12 mo.—being a biography of the most eminent Artists.

"Hard Scrabble," by Rev. ELIJAH KELLOGG; "Bear and Forbear," by OLIVER OPTIC; "The Boys of Grand Pré School," "The Little Maid of Oxbow," "The Pinks and Blues," by ROSE ABBOTT, illustrated; "Charley and Eva Roberts, or Home in the West." Then comes "The Proverb Stories," three volumes, in a beautiful case, illustrated. 1st. A Wrong Conferred, is Help Redressed. 2d. One Good Turn deserves Another. 3d. Actions speak Louder than Words. These are beautifully "got up," and no doubt will have a multitude of admiring readers among the boys and girls of America. Circular of information of the "Bureau of Education," from the Government Printing Office of Washington City, by JOHN EATON, JR., Commissioner, containing a great variety of Educational statistics, interesting to all Teachers and Professors in the numerous educational establishments in the country. Translation of an article by A. FICKER, Imperial Counsellor at Vienna, Austria—School Room Diseases, School Organization, &c.

"Protestantism and Presbyterianism, or the Retiring Moderator of the Southern General Assembly of 1870," which met in Louisville, Kentucky,

May 19, 1870. To outsiders, the necessity of such a publication will seem most unfortunate, and it is to be hoped that the day will come when all will be glad to let such things pass into oblivion.

MIDDLETON PUBLICATIONS.—"The Student's Mythology," a compendium of Greek, Roman, Egyptian, Assyrian, Scandinavian, Celtic, Aztec, and Peruvian mythologies, in accordance with standard authorities ; arranged for the use of schools and academies by C. A. WHITE. 315 pp., 12 mo., $150. This is a book which every well educated man will be glad to own as a standard reference—as useful twenty years hence as to-day.

"Wadsworth Sermons." We have seldom seen more impressive discourses than those from the pen of the great Philadelphia Presbyterian, delivered when he was Pastor of a San Francisco Church. "A Spectacle to Angels" is one of the sublimest discourses in print.

"Life of Arthur Tappen," by HURD & HOUGHTON. 432 pp. $2.00—a work which multitudes of educated men and woman cannot fail to read with absorbing interest.

"Hutchison's Physiology and Hygiene." 270 pp. 12 mo. By JOSEPH C. HUTCHISON, M. D. For the use of educational institutions and general readers. Published by Clark & Maynard, 5 Barclay Street, New York. $1.60. This work is well written, scientific and practical, and if read and heeded in every family in the land, would add largely to the average of human health, happiness and life.

The New Article of Food, "Sea Moss Farine," is pecularly adapted to winter. As a warming food, it promotes vital heat, and gives warmth from within outwards, the only healthy mode of securing a healthful and efficient circulation. It is a seasonable, healthy, and nutritious dessert.

"Wilson's Presbyterian Historical Almanac." The next volume will contain the Proceedings of the General Assemblies of 1870, together with the two preceding years, thus bringing all the Reunion Acts and Deliverances within the compass of a single volume, and will contain two hundred and sixty biographies of deceased Presbyterian Clergymen. Address in full, Joseph M. Wilson, 123 South Fourth Street, Philadelphia, Pa. Subscriptions in Great Britian and Ireland will be received by Trubner & Co., 60 Paternoster Row, London. Ten Shillings per copy. This is a labor of love for the Presbyterian Church, an inherited love on the part of Mr. Wilson, and merits the patronage of all the intelligent families of that body, besides that of the elders and clergy. The historical knowledge contained in the previous volumes becomes of increasing value every year, because in a permanent form. "The Physical Exploration of the Rectum, and the Ligation of Hemorrhoidal Tumors;" the method described by William Bodenhamer, A.M., M.D. Published by William Wood & Co., 31 Walker Street, New York—Sent from this office for one dollar. As Dr. Bodenhamer has been the most successful operator for Fistula and Piles in America, the medical profession will feel an interest in this publication not inferior to that for his previous published works.

